# Proteomics in Childhood Acute Lymphoblastic Leukemia: Challenges and Opportunities

**DOI:** 10.3390/diagnostics13172748

**Published:** 2023-08-24

**Authors:** Maria Kourti, Michalis Aivaliotis, Emmanouel Hatzipantelis

**Affiliations:** 1 Third Department of Pediatrics, School of Medicine, Aristotle University and Hippokration General Hospital, 54642 Thessaloniki, Greece; 2 Laboratory of Biological Chemistry, School of Medicine, Aristotle University of Thessaloniki, 54124 Thessaloniki, Greece; aivaliotis@auth.gr; 3 Children & Adolescent Hematology-Oncology Unit, Second Department of Pediatrics, School of Medicine, Aristotle University of Thessaloniki, 54124 Thessaloniki, Greece; hatzip@auth.gr

**Keywords:** acute lymphoblastic leukemia, proteomics, biomarkers

## Abstract

Acute lymphoblastic leukemia (ALL) is the most common cancer in children and one of the success stories in cancer therapeutics. Risk-directed therapy based on clinical, biologic and genetic features has played a significant role in this accomplishment. Despite the observed improvement in survival rates, leukemia remains one of the leading causes of cancer-related deaths. Implementation of next-generation genomic and transcriptomic sequencing tools has illustrated the genomic landscape of ALL. However, the underlying dynamic changes at protein level still remain a challenge. Proteomics is a cutting-edge technology aimed at deciphering the mechanisms, pathways, and the degree to which the proteome impacts leukemia subtypes. Advances in mass spectrometry enable high-throughput collection of global proteomic profiles, representing an opportunity to unveil new biological markers and druggable targets. The purpose of this narrative review article is to provide a comprehensive overview of studies that have utilized applications of proteomics in an attempt to gain insight into the pathogenesis and identification of biomarkers in childhood ALL.

## 1. Introduction

Acute lymphoblastic leukemia (ALL) is the most common cancer in children [1]. It is a biologically heterogeneous hematologic malignancy—mainly characterized by chromosomal alterations, and some somatic and genetic mutations—that leads to the dysregulation of cytokine receptors, hematopoietic transcription factors and epigenetic modifiers [2,3]. Contemporary chemotherapy for childhood ALL has resulted in a cure rate of more than 85% in developed countries, representing one of the success stories in treatment of childhood malignancies [4]. This can be attributed to: (i) risk-directed therapy, based on clinical features such as age and initial white blood count, (ii) biologic and genetic features such as karyotype and identification of cryptic translocations, but most importantly (iii) the response to treatment evaluation with minimal/measurable residual disease (MRD) [5,6]. However, despite the observed improvement in survival rates, leukemia remains one of the leading causes of cancer-related deaths [7]. Given the improved cure rates, current research has focused on subgroups of patients with refractory/relapsed disease. Identification of proteins and pathways related to cancer and its environment provides the potential to develop effective individualized treatment, especially in this high-risk group. Proteomics is a cutting-edge technique and a useful tool for creating innovative and customized therapy providing new prospects for precision-medicine strategies [8].

The purpose of this narrative review article is to provide a comprehensive overview of published studies that have utilized applications of proteomics in an attempt to gain insight into the pathogenetic pathway and biomarkers discovery in childhood ALL. A thorough computerized search of the PubMed/Medline database was carried out along with research material from conference proceedings, publications from American Society of Hematology, European Hematology Association, and book chapters. Literature search on PubMed/Medline database was conducted, referring to manuscripts/studies published between 1 January 2010 and 31 January 2023. The last search was carried out on 30 March 2023 with the following keywords: “pediatric” or “children” or “childhood” AND “acute lymphoblastic leukemia” or “ALL” AND “proteomic profile” or “proteomics” or “proteome”. Search results were narrowed down to studies published in the English language. Exclusion criteria included studies exclusively conducted in adults, studies involving conditions other than ALL as well as abstracts without full-text articles. Relevance and duplicates were primarily assessed based on the title and abstract screening.

The review comprises three relevant sections: an overview on the molecular basis of childhood ALL, an overview on proteomic analysis and a discussion of the applications of proteomics research in childhood ALL.

## 2. Molecular Basis of Acute Lymphoblastic Leukemia

Acute lymphoblastic leukemia is a hematologic malignancy of lymphoid origin and the most common childhood cancer representing about 25% of cancer diagnoses [9]. The highest peak age of incidence is between two and five years [10]. It is more frequent in boys than in girls with an approximate ratio of 1.3:1. Children of Hispanic descent are more frequently affected followed by White, and to a lesser percentage, African Americans [11]. Classification, based on immunophenotype, consists of 80–85% B-cell and 15–20% of T-cell, increasing in adolescence. Leukemic cells initiate in the bone marrow (BM) and infiltrate extramedullary sites such as the liver, spleen, mediastinum and lymph nodes and also sanctuary sites, such as the central nervous system (CNS), ovaries in girls and testes in boys.

Although environmental, immunologic, socioeconomic and epidemiologic factors have been evaluated rigorously as contributing factors to leukemogenesis, the exact underlying etiology remains unknown [12]. Before the advent of next-generation sequencing (NGS), only a small number of uncommon constitutional leukemia predisposition syndromes, such as Down syndrome (DS) and Li–Fraumeni syndrome, were associated with the development of ALL [13]. Other genetic syndromes linked to an increased risk of ALL include: Bloom syndrome, ataxia telangiectasia (AT), neurofibromatosis 1 and constitutional mismatch repair deficiency (CMMRD) [13]. The latter, and AT, have a preponderance to T-ALL, while B-ALL occurs almost exclusively in DS-ALL [14,15]. During the last decades, the landscape of germline and somatic mutations in childhood ALL has been unveiled with the aid of a microarray analysis of gene expression and ultra-high throughput sequencing technologies. Based on genomic analysis, childhood ALL is subdivided into genetic subgroups [16,17,18].

Gross chromosomal alterations in childhood ALL have been associated with outcome. Fluorescence in situ hybridization (FISH) assays and cytogenetics are used to identify structural chromosomal gains or losses in leukemic cells. High-hyperdiploidy (>50 chromosomes: 51–67 chromosomes) with trisomies of chromosomes 4,6,10,14,17,18,21, X occurs in almost 25% of childhood ALL cases and is associated with excellent outcome, even with reduced intensity chemotherapeutic regimens [19,20]. Low-hyperdiploidy with 47–50 chromosomes was traditionally associated with a poor outcome. However, contemporary therapy regimens have significantly improved clinical outcome [21,22]. On the contrary, hypodiploid B-ALL with less than 44 chromosomes is uncommon and associated with a poor outcome, especially in children with positive minimal residual disease at the end of induction. Germline *TP53* mutations consistent with Li–Fraumeni syndrome occur frequently in low-hypodiploid ALL subtype [23,24].

Chromosomal translocations represent a molecular hallmark of childhood ALL and represent a significant prognostic factor. Chimeric fusion genes are created by chromosomal rearrangements and involve epigenetic modifiers, tyrosine kinases and transcription factors [25]. They can be identified with reverse-transcription polymerase chain reaction amplification of the fusion genes created, together with FISH assays. Routine cytogenetic analyses fail to detect some of the cryptic translocations. Almost one fourth of standard-risk B-ALL harbor the cryptic t(12;21)(p13;q22), resulting in ETV6-RUNX1 (TEL-AML1) fusion. It should be noted that this fusion can also be detected in children who do not develop leukemia. It has also been detected in preserved blood spots from children who later develop ALL indicating a potential prenatal origin of leukemogenesis in association with additional necessary co-operating mutations for the development of leukemia [26]. A small group of “ETV6-RUNX1-like” B-ALL has also been reported. These cases lack the classic ETV6-RUNX1 rearrangement and are associated with other ETV6 fusions and with IKZF1 deletions. A unifying and prominent feature of a majority of prognostic studies in pediatric BCP ALL is that the different types of IKZF1 deletions have been constantly linked to an unfavorable clinical outcome of frontline treatment [27]. Most children with ETV6-RUNX1 fusion belong to the standard risk group and exhibit excellent outcome with standard therapy [21].

Other common rearrangements in B-ALL include: t(1;19)(q23;p13.3) resulting in TCF3-PBX1 (E2A-PBX1) fusion, rearrangement of KMT2A (formerly MLL; 11q23), and t(9;22)(q34;q11.2) (the Philadelphia chromosome) resulting in BCR-ABL1 fusion. While TCF3-PBX1 ALL was formerly linked to an intermediate or unfavorable prognosis, modern therapeutic regimens have enhanced outcome; thus, TCF3-PBX1 fusion is no longer considered for risk stratification [28]. Children with TCF3-PBX1 ALL appear to have higher risk of CNS relapse and may warrant intensification of CNS-directed therapy [29]. TCF3-HLF fusion resulting from t(17;19)(q22;p13.3) is very uncommon in patients with B-ALL and, despite its rarity, has been associated with extremely poor outcome [30]. MLL rearrangement involving somatic translocations of the KMT2A gene are common in infant B-ALL. Outcome is generally dismal in infants aged less than 3 months, and clinical prognosis varies according to the specific KMT2 translocation [31]. A trial conducted in KMT2A rearranged infant ALL investigating incorporation of lestaurtinib, an FLT3 inhibitor, with intensive chemotherapy, based on the FLT3 overexpression, revealed that addition of lestaurtinib did not improve outcome [32,33].

An established molecular-targeted therapy paradigm in childhood ALL is Philadelphia positive (Ph+) ALL with the t(9;22)(q34;q11) resulting in BCR-ABL1 oncoprotein. Three BCR-ABL1 protein isoforms are encoded, the p210, the p190, and the p230, which have persistently enhanced tyrosine kinase (TK) activity. The BCR-ABL fusion gene of childhood and adult ALL have a different molecular basis, with the BCR-ABL fusion gene in adult ALL of the “p210” subtype resembling that found in chronic myeloid leukemia (CML), whereas the childhood subtype is mainly “p190” [34]. Historically, the best curative option has been hematopoietic stem cell transplantation (HSCT). The addition of tyrosine kinase inhibitors (TKIs) in the intensive chemotherapy backbone, has significantly improved event-free and overall survival [35,36]. BCR-ABL1-Like or Philadelphia chromosome-like ALL has recently been described as a subset of B-ALL defined by an activated kinase gene expression profile similar to that of Ph+ ALL and is associated with miscellaneous genetic alterations that activate cytokine receptor signaling pathways [37]. In spite of the heterogeneity in Ph-like kinase-activating alterations, JAK-STAT, ABL, Ras/MAPK signaling pathways are activated and can effectively be inhibited by relevant TKIs [38]. The Janus kinase-signal transducer and activator of transcription (JAK-STAT) pathway plays a major role in transmitting signals from cell-membrane receptors to the nucleus leading to important physiological processes such as immune regulation, hematopoiesis, cell proliferation and survival [39]. The mitogen-activated protein kinase (MAPK) pathway, consisting of the Ras-Raf-MEK-ERK signaling cascade, is a crucial cellular network that regulates apoptosis, cellular development, differentiation, and proliferation [40]. Some studies have revealed a functional interaction between the JAK/STAT and RAS/MAPK pathways that promotes leukemogenesis and uncontrolled cell proliferation [41].

In contrast to B-ALL, the identified genetic alterations that occur in T-ALL do not add a substantial prognostic value in the established risk stratification of T-ALL based on CNS and MRD status [42]. Mutation of TAL1 (1p32) is a non-random genetic defect frequently present in childhood T-ALL. The SIL/TAL1 fusion product gives rise to inappropriate expression of TAL1, that may promote T-cell leukemogenesis. The clinical relevance and the prognostic value of this rearrangement remains to be further elucidated [43]. The most common dysregulated signaling pathway is the Notch pathway, either by upregulation with activating mutations of Notch1 or by loss of function of negative regulators such as FBXW7 [44]. Although gamma-secretase inhibitors (GSI) seem promising in the inhibition of the Notch pathway, the gastrointestinal toxicity and lack of efficacy prohibited their use in T-ALL therapy [44]. Interestingly, early T-cell precursor ALL shows JAK-STAT and Ras pathway mutations and is characterized by a distinct gene signature of the JAK-STAT signaling pathway that might explain the chemoresistance of T-ALL. Nevertheless, it can be inhibited by pathway inhibitors in leukemia models [45]. The nucleoside analogue nelarabine has proven to show early activity in patients with relapsed/refractory T-ALL, but it was associated with neurotoxicity [46]. The proteasome inhibitor, bortezomib was examined in the Children’s Oncology Group phase III clinical trial AALL123, in which only patients with T-lymphoblastic lymphoma showed a significantly improved survival [47].

The most common translocations in T-ALL involve fusion of T-cell receptor genes. Gene expression signatures define novel oncogenic pathways in T-cell ALL, but their prognostic significance remains unidentified. Although stimulating advances have occurred regarding the genomic characterization of T-ALL, development of precision medicine treatment approaches for T-ALL has proven more challenging [48].

Approximately 20% of pediatric ALL patients experience a relapse with a survival lagging behind newly diagnosed ALL. Major pathways of lymphoid development, kinase signaling, cell cycle regulation and epigenetic modification are involved in the genetic basis of relapsed ALL [49]. Molecular-targeted therapy has shown promising results in early trials, though not translated into improved survival [50,51].

Relapsed and refractory disease pose extreme therapeutic challenges, demonstrating an unmet need for the development of durable therapies. Recent advances in immunotherapy with CD19 inhibitors and CD22 inhibitors (Blinatumomab and Inotuzumab ozogamicin, respectively) and chimeric antigen receptor (CAR)-T cell therapy, have reformed the management of relapsed and refractory ALL with the price of serious adverse events, such as cytokine release syndrome, immune effector neurotoxicity syndrome, prolonged BM suppression and hypogammaglobulinemia [52]. They offer the advantage to act independently of genetic aberrations and overcome drug-resistant mutations enhanced in relapsed ALL [53]. Therefore, these therapies seem ideal for patients who do not express targetable genetic alterations. Whether these immunotherapies could serve as definite therapies rather than bridging therapies to HSCT remains to be elucidated in the near future.

## 3. The Era of Proteomics

Proteomics studies the structure along with the function of the proteome [54]. The term proteome describes the functional state of the total of proteins, which are responsible for the functional activity of different cells [55]. As a result, study of the proteome correlates the structural and functional multiplicity of proteins during the disease process. The field of proteomics has been developed since genomics is ineffective in unravelling the structure and dynamic state of proteins, which are gene products [56]. In contrast to the steady state of the genome, expression of a protein reflects a dynamic state of processes including RNA transcription, alternative splicing, and/or post-translational modifications (PTM) [57]. PTMs have fundamental regulatory properties, such as converting a protein from its inactive to its active state, or determining a protein’s half-life resulting from ubiquitination or acetylation; hence, defining its functional property in a cell and tissue-specific context that ultimately determines the resulting cellular phenotype and its biological significance [58]. Regulation of protein translation, degradation and PTM lead to low association between the cellular proteome and genome/transcriptome [59]. As a result, phenotypical and functional changes are not frequently visible at the genome level but apparent in the proteome.

The general strategy pursued in proteomics is to compare related samples from different disease stages considering that differences in their proteome could reflect a different disease stage. Proteomic studies are carried out mainly in body fluids such as the cerebrospinal fluid (CSF), peripheral blood (PB) and BM. Body fluids exhibit a great emerging potential for biomarker studies, in particular those that can be collected by minimally invasive techniques [60,61]. The high potential of serum/plasma as a source for protein biomarkers is reflective of the overall state of an organism [62,63,64]. Alternatively, cancer cell lines or patient-derived tumors can also be engrafted into immunocompromised mice to generate the so-called cell line-derived xenografts (CDX) or patient-derived xenografts (PDX), respectively [65,66,67].

**Figure 1:**
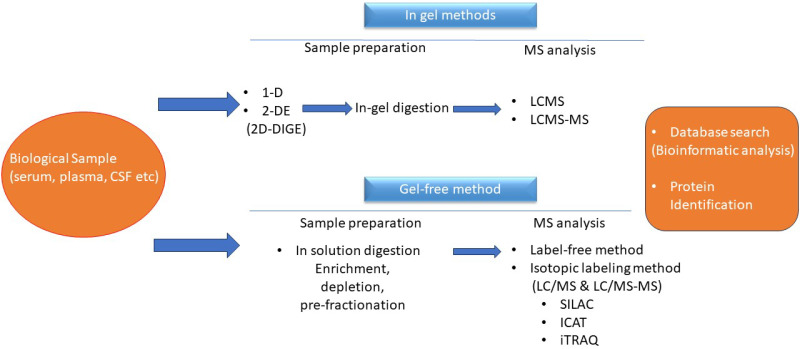
Workflow for proteomic analysis.

Biomarkers are recognized using mass spectrometry (MS) [68]. Mass spectrometry requires a low-energy ionization source that transfers peptides from solid/liquid to gaseous states (matrix-assisted laser desorption/ionization, MALDI, and electrospray ionization, ESI). The two basic and commonest types of mass analyzers are time of flight (ToF) and ion trap resonance analyzers. Computer algorithms help vastly in the immense task of identifying peptides and ultimately the proteins from which they are derived. Computational methods and statistical algorithms can maximize the mining of proteomic data. Quantification MS-based methods are divided into the label-free and stable isotope label approach. In the label-free approach, individual samples are injected directly in the MS and the relative abundance of peptides is quantified [69]. The major advantage of this technique is minimal sample handling, but problems in reproducibility and accuracy are encountered [69]. Furthermore, the extensive instrument time required is a disadvantage for the large sample sets typical for biological and clinical studies. On the contrary, a stable isotope labeling strategy, such as an isobaric tag, allows for the mixing of multiple samples at different stages [70]. A workflow of proteomics is depicted in Figure 1.

There are two different methodological approaches in proteomics. The first, known as top-bottom, is the discovery of a protein through its selective isolation, the characterization of its sequence and structure and the study of function, regulation, interaction and PTM. The second is the bottom-up or the so-called “shotgun” aiming to identify all the proteins present in a sample. In shotgun proteomics, which is a gel-free liquid chromatography (LC)-MS approach, it is essential to know a protein’s identity before quantification, as peptides need to be related to each other and to their parent proteins; only then is protein quantification possible. Therefore, gel-based proteomics usually identifies only proteins with different abundances, while LC-MS identifies all detected proteins [71]. Currently, most of proteome analysis is performed with label and label-free shotgun proteomics [72].

## 4. Discussion—Application of Proteomics in Childhood ALL

Since the hallmark of ALL is the uncontrolled clonal proliferation of poorly differentiated lymphoid progenitor cells inside the bone marrow, interfering with the production of blood cells, serum and plasma may serve as rich sources of blood cancer-associated biomarkers. 

In an attempt to determine potential disease markers in childhood B-ALL, a Colombian exploratory study group performed a proteomic study implementing LC-MS/MS and quantification by label-free methods searching for proteins differentially expressed be-tween healthy children and children with B-ALL. They quantified 472 proteins in depleted blood plasma and found that 25 proteins were differentially expressed [73].

Moreover, differential proteins were analyzed by MALDI-TOF-MS and were identified in lymphocytes in patients with childhood ALL and healthy children, by Wang et al. [74]. Among the 25 differential proteins, eight provided a valuable insight into the molecular mechanism of leukemogenesis and could serve as candidate markers or drug targets. Cellular levels of GSTP in c-ALL samples were dramatically up-regulated and may be regarded as a biomarker and drug target together with PHB that was also up-regulated, suggesting that it might be associated with leukemogenesis [74].

Candidate biomarkers for early diagnosis of B-ALL were overexpressed in a proteomic analysis with lectin affinity chromatography LC-MS of serum from pediatric patients with B-ALL performed by Cavalcante et al. [75]. A total of 96 proteins were identified and among them leucine-rich alpha-2-glycoprotein 1 (LRG1), clusterin (CLU), thrombin (F2), heparin cofactor II (SERPIND1), alpha-2-macroglobulin (A2M), alpha-2-antiplasmin (SERPINF2), Alpha-1 antitrypsin (SERPINA1), complement factor B (CFB) and complement C3 (C3) were identified as candidate biomarkers for early diagnosis of B-ALL, as they were up-regulated in the B-ALL group compared to controls after induction therapy [75].

Identification of tumor autoantibodies may be utilized in early cancer diagnosis and immunotherapy. Serological proteome analysis (SERPA) is another proteomic approach and screening autoantibodies as serum biomarkers of B-ALL using SERPA with combination of 2-DE, immunoblotting and MS revealed that α-enolase and VDAC1 autoantibodies were promising biomarkers for children with B-ALL. Evaluation of serum autoantibodies against α-enolase and VDAC1 show promising clinical applications [76].

Braoudaki et al. [77]. evaluated the differential expression detected in the proteomic pro-files of pediatric low- and high-risk ALL patients aiming to characterize candidate biomarkers related to diagnosis, prognosis, and patient-targeted therapy. Proteomic analysis was performed using 2DE and protein identification by MALDI-TOF-MS and revealed that CLUS, CERU, APOE, APOA4, APOA1, GELS, S10A9, AMBP, ACTB, CATA and AFAM proteins play a significant role in leukemia prognosis, potentially serving as distinctive biomarkers for leukemia aggressiveness, or as suppressor proteins in HR-ALL cases. Moreover, bicaudal-D-related protein 1 (BICR1) could probably serve as a significant biomarker for pediatric ALL therapeutics [77].

Xu et al. [78]. performed proteomic analysis comparing the differentially expressed proteins between high-risk and low-risk childhood B-ALL by a label-free quantitative proteomics [78]. In the high-risk childhood B-ALL, 86 differently expressed proteins were depicted, and 35 proteins were predicted to have directive interactions. They found that, in high-risk B-ALL, the aberrant events might happen in pre-mRNA splicing, DNA damage response, and stress response contributing to the high-risk classification [78].

Jiang et al. [79]. using proteomic tools (2DE coupled to MS) in clinically important leukemia cell lines (REH, 697, Sup-B15, RS4.11), together with bone marrow samples from children with ALL, identified potential prognostic protein biomarkers and promising regulators of PRED-induced apoptosis. In patients with a good response to prednisone, down-regulation of PNCA was identified, while in prednisone poor responders (PPRs), proteins remained unchanged [79].

Hu et al. [80]. hypothesized that identification of proteins through proteomic analysis might lead to novel insights into resistance mechanisms in acute leukemia [80]. They applied proteomics tools via DIGE followed by MALDI-TOF, aiming to study the expression difference of cellular proteins between the drug-sensitive HL-60 and adriamycin-resistant HL-60 (HL-60/ADR) cell lines. They found that the up-regulations of nucleophosmin/B23 (NPM B23) and nucleolin C23 (C23) could be related to resistance of leukemia and could provide an important prognostic leukemia indicator [80].

Resistance to chemotherapeutics used in ALL is a major challenge and involves com-plex cellular processes. Guzmán-Ortiz et al. [81] studied proteome changes in B-lineage pediatric ALL cell line CCRF-SB after adaptation to vincristine, a vinca alkaloid used in ALL therapy [81]. Vincristine, by interaction with tubulin, disrupts the microtubule polymerization, resulting in cell cycle arrest and apoptosis [82]. They found 135 proteins exclusively expressed in the presence of vincristine, indicating that signal transduction and mitochondrial ATP production may serve as potential therapeutic targets. In a previous study, Verrills et al. [83]. reported the proteomic changes of the T-lineage ALL cell line CCRF-CEM after exposition to vincristine. They found that vincristine induced changes in the expression of 39 proteins and that a resistant subline differentially expressed 42 proteins mainly involved in cytoskeleton metabolism regulation of apoptosis, gene, chaperones and ribosomal proteins [83].

Leukemia studies aim to decipher the mechanisms, pathways and the degree to which the proteome impacts leukemia subtypes and to identify whether disease stratification based on proteome features could provide precise targets for therapy. Strategies of phosphoproteomics can be used to profile the activation/deactivation of crucial molecules in signaling pathways which are key to the progression, remission and relapse of leukemia, since leukemogenesis is controlled via the regulation and interaction of signaling cascades [84,85]. A commonly mutated pathway in pediatric cancers is the receptor tyrosine kinase/ras (RTK/RAS) pathway. Mutations in this pathway are identified as possible targets for treatment and are mainly implicated on clonal evolution in high hyperdiploid ALL, a subtype of the most common childhood cancer [86,87]. Mutations of KRAS in signal transduction domains considerably affect the ability of proteins to accomplish their normal cell-signaling functions [87]. In a study by Siekmann et al. patient-derived xenograft ALL (PDX-ALL) models were established with dependencies on fms-like tyrosine kinase 3 (FLT3) and platelet-derived growth factor receptor b (PDGFRB), which were interrogated by phosphoproteomics using iTRAQ mass spectrometry [88]. Phosphoproteomic analyses identified group I PAKs as targets of RTKs in ALL, while PAK inhibition affects in vitro growth and survival of ALL cells [88]. According to the findings of this study, PAKs is identified as a potential downstream target in RTK-dependent childhood ALL, the inhibition of which might assist in preventing the selection or acquisition of resistance mutations toward tyrosine kinase inhibitors [88].

The JAK/STAT pathway have also been implicated in the oncogenesis of many cancers as well as of childhood leukemia [89]. A JAK mutant has been identified in childhood B-cell ALL leading to overactivity in cell proliferation [89]. Somatic mutations in tyrosine-protein phosphatase non-receptor type 11 (PTPN11) lead to hyperactivation of the catalytic activity instead of the normal inhibitory function [90,91]. The Notch signaling pathway is one of the most frequently overactivated signaling pathways in cancer, and mutations in Notch family proteins are detected in a majority of T-cell ALL [92]. Activation of γ-secretase is crucial in the activation of the Notch pathway and inhibitors can be applied to block this activation. These are currently tested in clinical trials [93]. Increased mTOR activity has been implicated in ALL relapses and has been suggested as a therapeutic candidate target [94,95]. The crucial function of proteins on the stimulation of signaling pathways in the course of diagnosis and predicting relapse remains to be elucidated [96]. Knowledge of the proteomic landscape of ALL that emerges from the consequences of genetic and epigenetic events would prove to be valuable in identifying “druggable” target proteins.

High hyperdiploid B-cell ALL and ETV6/RUNX1-positive pediatric ALL are among the most common subtypes of childhood leukemia. In a study by Yang et al. [97]. it has been demonstrated that the characteristic extra chromosomes have an impact on the transcriptome and proteome suggesting that hyperdiploid leukemia cases harbor aberrant chromatin organization that causes genome-wide transcriptional dysregulation [97]. In hyper-diploid cases, 1286 proteins were up-regulated (more important of which were CD44 and FLT3) and 1127 were down-regulated (IGF2BP1 CLiC5 RAG1 RAG2) by proteome HiRIEF LC-Ms/MS analyses. A previous study by Costa et al. [98]. on protein expression in pre-B2 lymphoblastic cells from children with ALL in relation with t(12;21) translocation has identified several proteins of interest, defining a “protein-map” associated to some sub-groups of patients with particular features. The correlation between the proteins’ expression and the t(12;21) or its fusion transcript ETV6-RUNX1 still remains to be verified. Nevertheless, this new approach for identification and classification of patient subgroups could lead to interesting therapeutic targets [98].

Children with T-ALL display resistance in glucocorticoid treatment. Serafin et al. identified, by reverse-phase protein arrays, that lymphocyte cell-specific protein-tyrosine kinase (LCK) was aberrantly activated in PPR patients [99]. They also showed that LCK inhibitors, such as dasatinib, bosutinib, nintedanib, and WH-4-023, could induce cell death in GC-resistant T-ALL cells, and remarkably, co-treatment with dexamethasone is capable of reversing GC resistance, even at therapeutic drug concentrations. These results offer a new insight into the biology and treatment of pediatric T-ALL by providing a new targeted therapy option with the use of LCK inhibitors, which could be easily rendered into clinical practice in an attempt to overcome GC resistance and improve the outcome of poor-responder T-ALL pediatric patients [99].

Phosphoproteomics is designed to provide information on pathway activation and signaling networks and offer opportunities for targeted therapy [100]. In a recent MS-based global phosphoproteomic profiling of 11 T-cell ALL cell lines targetable kinases were recognized [101]. Cordo et al. [100]. reported a comprehensive dataset consisting of 21,000 phosphosites on 4896 phosphoproteins, including 217 kinases. Moreover, they identified active Src-family kinases signaling and active cyclin-dependent kinases. They validated putative targets for therapy ex vivo and detected potential combination treatments, such as the inhibition of the INSR/IGF-1R axis to increase the sensitivity to dasatinib treatment. Moreover, since multiple clinical trials are investigating the JAK inhibitor ruxolitinib for the treatment of T-ALL in the presence of JAK mutations, Cordo et al. [100]. showed that ruxolitinib treatment is effective ex vivo in T-ALL cells with elevated JAK kinase activity. Mass spectrometry-based phosphoproteomic studies dedicated to T-ALL may prove to be helpful tools to decipher specific pathological signaling pathways and escape mechanisms. This will ultimately lead to the identification of novel disease-specific or individualized biomarkers [102].

Studies on the comparative analysis between the bone marrow tumor microenvironment and the peripheral blood are very important to decipher the ALL biology. Overarching challenges of the hidden mechanisms behind immature lymphoid cells accumulation in the bone marrow and the mechanisms underlying the chemotherapy effects against B-ALL will shed light on the leukemia biology and the cumulative impact of chemotherapy [103]. A recent study by Brotto et al. [103] highlighted a possible role for transthyretin and IFN-g as mechanisms related to disease remission.

Noteworthy, interesting results were depicted in a recent study by Leo et al. [104]. who performed a comprehensive multi-omic analysis of 49 readily available childhood ALL cell lines, using proteomics, transcriptomics, and pharmacoproteomic characterization. They connected the molecular phenotypes with drug responses to 528 oncology drugs, identifying drug correlations as well as lineage-dependent correlations [104]. Their observations indicate that both conventional lineage and oncogenic traits contribute to proteome-level differences in their cell line panel. Phenotypic profiling supports current clinical practice in leukemia stratification and suggests that MS-based proteomics could be an effective path to discover the drivers contributing to pathogenic phenotypes. They identified the diacylglycerol-analog bryostatin-1 as a therapeutic applicant in the MEF2D-HNRNPUL1 fusion high-risk subtype, for which this drug triggers pro-apoptotic ERK signaling linked to molecular mediators of pre-B cell negative selection [104]. Table 1 summarizes proteomic studies on childhood ALL.

Central nervous system involvement remains one of the major causes of ALL treatment failure [105,106]. Despite the therapeutic advances in ALL, CNS relapse occurs in 3–8% of the children with ALL and is associated with increased morbidity and mortality [107]. In normal physiological conditions, 80% of the protein in the CSF is hematogenic in origin [108]. Thus, protein content changes in CSF provide an attractive approach to study hematological malignancies [109]. Although CSF is obtained by using an invasive method, it is considered as the optimal fluid for diagnosis of CNS infiltration in ALL. Efforts have been made in recent years to detect novel biomarkers of hematologic malignancy in CSF [110]. As demonstrated in a pilot study, gel-free, label-free quantitative proteomics is feasible for profiling of CSF in pediatric leukemia/lymphoma [74,111]. Moreover, in the same study, the expression of antithrombin III and plasminogen decreased over time in one child who developed CNS thrombosis, compared to other subjects [111]. In another prospective pilot study, quantitative proteomics by using LC-MS/MS was used to discover differential expression of CSF protein in newly diagnosed children with leukemia and CNS infiltration versus healthy controls aiming to discover possible prognostic biomarkers [112]. Using LC-MS/MS, 51 proteins were identified to be significantly different between the two groups including 32 proteins that were up-regulated and 19 proteins that were down-regulated. Among them, TIMP1, LGALS3BP, A2M, AHSG, FN1, HRG, and ITIH4 have been associated with cancer, while CF I, C2, and C4A, have been related to complement activity [112]. In another study, Mo et al. implemented label-free LC−MS/MS in order to explore proteomic profiles in patients suffering from ALL with CNS infiltration [113]. Among the 428 unique proteins identified, they quantified 10 altered proteins during treatment [113].

Some of the proteins are likely to play a vital biological role as biomarkers for the development of ALL, with diverse biological functions after induction chemotherapy. It is noteworthy that those altered proteins should be further investigated as predictive markers of ALL with CNS infiltration, some of which may have the prospect of becoming new therapeutic targets in childhood ALL with CNS involvement. Interesting results have been reported from an exploratory study by Yu et al. [71]. who investigated protein dynamics in children with B-cell ALL undergoing chemotherapy by implementing a 4-plex N,N-dimethyl leucine isobaric labeling strategy in a longitudinal study [71]. According to their results, neural cell adhesion molecule (NRCAM), neuronal growth regulator 1, (NEGR1) and secretogranin-3 (SCG3,) were significantly altered at different stages of chemotherapy. All the aforementioned proteins have been related to neurologic disorders and may reflect CNS injury caused by chemotherapy on neuronal membranes and other structures. Thus, they may also be implicated in long-term neurocognitive effects of chemotherapy [71]. Table 2 summarizes the proteomic studies conducted in CSF in relation to ALL.

## 5. Conclusions

Childhood ALL is a biologically heterogeneous disease characterized by structural alterations, genetic and somatic mutations through a process of complex protein-based signaling network pathway modifications. Changes in protein expression can partially be determined by the analysis of the static genome. Although implementation of next-generation genomic, transcriptomic, and epigenetic sequencing tools unveiled the genetic landscape of childhood ALL, uncovering the underlying dynamic changes at a protein level still remains a challenge.

Proteomics offers complementary information to genomics and transcriptomics by analyzing protein structure, expression, and modification status. Advances in the field of MS enables high-throughput collection of global proteomic profiles representing an opportunity to discover new biological markers and druggable targets. Novel disease-specific biomarkers will provide an additional reference for predicting treatment responses and individual prognosis. Deeper characterization of biomarkers and molecular pathways, especially in patients with relapsed/refractory disease, will ultimately identify precision medicine candidates for application of translational precision therapy.

## Figures and Tables

**Table 1:** Synopsis of proteomic studies of childhood ALL.

Author/Year/Country	Objective	Sample	Proteomic Approach	Main Results
Jiang N et al. [79] 2011 Singapore	Identify potential prognostic protein biomarkers. Discover promising regulators of PRED-induced apoptosis	Cell Line Bone marrow	2-DE & MALDI-TOF	PCNA was highly predictive of PRED response in patients independent of molecular subtype.
Braoudaki M et al. [77] 2013 Greece	Evaluate differential expression in proteomic profiles of low risk- and high risk-ALL. Characterize candidate biomarkers related to diagnosis, prognosis and patient targeted therapy	Plasma Bone marrow	2-DE & MALDI-TOF	CLUS, CERU, APOE, APOA4, APOA1, GELS, S10A9, AMBP, ACTB, CATA and AFAM proteins play a role in leukemia prognosis Vitronectin and plasminogen: contribute to leukemogenesis. Bicaudal D-related protein 1: biomarker for pediatric ALL therapeutics.
Wang D et al. [74] 2013 China	Compare differential proteins in lymphocytes between children with c-ALL and healthy children. Explore the mechanisms of c-ALL. Find new diagnostic and therapeutic strategies for c-ALL.	Bone marrow	SDS-PAGE MALDI-TOF	15 proteins differentially expressed. 2 high expressions (Glutathione S-transferase P, PHB) 6 low expressions (PRDX4, 60s acidic ribosomal protein P0, Cytoplasmic actin,pyridoxine-5′-phosphate oxidase, Triosephosphate isomerase 1, FLJ26567)
Costa O et al. [98] 2014 France	Compare the lymphoblastes proteome in c-ALL in accordance with the presence of t(12;21)	Bone marrow	2-DE nanoLC Ion trap MS/MS	Over-expression of CNN2, MAT-2β, hnRNPA2, PITPβ, PSMB2, HSPC263 (OTUB1). Under-expression: BUB3, hnRNPE2, PSMB6, CK2a
Cavalcante MS et al. [75] 2016 Brazil	Perform proteomic analysis of serum from pediatric patients with B-ALL Identify candidate biomarker proteins, for use in early diagnosis and evaluation of treatment.	Serum	FPLC nanoUPLC-ESI-MS/MS	Upregulation and candidate biomarkers for early diagnosis: Leucine-rich alpha-2-glycoprotein 1 (LRG1), Clusterin (CLU), thrombin (F2), heparin cofactor II (SERPIND1), alpha-2-macroglobulin (A2M), alpha-2-antiplasmin (SERPINF2), Alpha-1 antitrypsin (SERPINA1), Complement factor B (CFB) and Complement C3 (C3).
Xu G et al. [78] 2017 China	Figure out the critical altered proteins which can indicate the risk rank	Bone marrow	LC-ESI-MS/MS	86 differently expressed proteins in the high-risk B-ALL 35 proteins have directive interactions
Guzmán-Ortiz AL et al. [81] 2017 Mexico	Identify changes in the proteome after adaptation to vincristine.	B-ALL cell line	MS ESI-MS/MS	135 proteins exclusively expressed in the presence of vincristine—represent potential therapeutic targets (Toll receptor signaling pathway, Ras Pathway, B-T cell activation, CCKR signaling map cytokine-mediated signaling pathway, oxidative phosphorylation.)
Serafin V et al. [99] 2017 Italy	Identification of deregulated signaling pathways to point out new targeted approaches.	Bone marrow Cell lines Primary xenograft (PDX) cells	Reverse-phase protein arrays (RPPA)	Lymphocyte cell-specific protein-tyrosine kinase (LCK): aberrantly activated in PPR patients. Resistance to glucocorticoid treatment in pediatric T-ALL can be reversed by LCK inhibitors in vitro and in vivo. IL-4 overexpression contributes to LCK-induced glucocorticoid resistance.
Siekmann IK et al. [88] 2018 Germany Canada UK	Decipher signaling circuits that link RTK activity with biological output in vivo	Patient-derived xenograft ALL (PDX-ALL) models with dependencies on fms-like tyrosine kinase 3 (FLT3) and platelet-derived growth factor receptor b (PDGFRB)	Phosphoproteomics iTRAQ nano-LC MS/MS	Group I PAKs act as signaling hubs in RTK-dependent pathways in ALL. Inhibition of group I PAKs by FRAX486 augments the antileukemic efficacy of midostaurin in FLT3-driven ALL.
Calderon-Rodrıguez SI et al. [73] 2019 Colombia	Study of the plasma proteome of Colombian children diagnosed with B-cell ALL (B-ALL) to determine potential disease markers that reflect processes altered by the presence of the disease or in response to it.	Plasma	Nano-LC-MS/MS	25 proteins were differentially expressed in B-ALL Potential biomarkers that could be used to differentiate unhealthy patients from healthy individuals
Yang M et al. [97] 2019 Sweden Germany UK	Proteogenomic analysis of a series of pediatric BCP-ALL, including high hyperdiploid and diploid/near-diploid ETV6/RUNX1-positive cases, aiming to determine the effects of aneuploidy.	Bone marrow	HiRIEF LC-MS/MS	Proteins differentially expressed between hyperdiploid and ETV6/RUNX1-positive leukemia had higher mRNA-protein correlations. CTCF and cohesin: low expression in hyperdiploid ALL
Broto GE et al. [103] 2020 Brazil	Comparing bone marrow and peripheral blood profiles, before chemotherapy—at diagnosis, and the cumulative effects found after the induction treatment	Bone marrow Peripheral blood B-ALL	Nano-LC-MS/MS	D0, PB characterized as a pro-inflammatory environment, with the involvement of several down-regulated. Coagulation proteins as KNG, plasmin, and plasminogen. D28 characterized by immune response-related processes and the super expression of the transcription factor IRF3 and transthyretin. RUNX1 found in both D0 and D28. IRF3-IFN γ axis induction as a possible mechanism enrolled in disease resolution, transthyretin as an up-regulated protein induced by the induction phase of chemotherapy.
Uzozie A et al. [96] 2021 Canada	Reveal proteome signatures characteristic of leukemic subtypes. Explore how effectively PDX models replicate the primary leukemic proteome.	Cell lines Bone marrow Peripheral blood PDXs	LC-MSMS	Proteome differentiates pediatric B-ALL and T-ALL from non-leukemic cells. The proteome of xenograft ALL closely resemble matched patient proteome. Relapse specific changes in patients are retained in PDXs. Proliferation and immune response processes differ between PDXs and patients. Xenografts recapitulate proteome response to structural genomic changes in patients.
Cordo V et al. [100] 2022 Netherlands	Provide information on pathway activation and signaling networks that offer opportunities for targeted therapy.	T-cell ALL lines PDXs	Reverse phase protein array (RPPA)	21,000 phosphosites on 4896 phosphoproteins, including 217 kinases. Validate putative targets for therapy ex vivo and identify potential combination treatments, such as the inhibition of the INSR/IGF-1R axis to increase the sensitivity to dasatinib treatment.
Yu R et al. [76] 2022 China	Screen serum autoantibodies of pediatric B-ALL, aiming to contribute to the early detection of B-ALL in children.	Pooled B-ALL cell lines (NALM-6, REH and BALL-1 cells)	Serological proteome analysis (SERPA)	α-enolase and VDAC1 were identified as candidate autoantigens in children with B-ALL and potential serological markers.
Leo IR et al. [104] 2022 Sweden	Perform comprehensive multi-omic analyses using proteomics, transcriptomics, and pharmacoproteomic characterization	Childhood ALL cell lines	LC-MS	In-depth proteomic analysis of childhood ALL cell lines covering numerous cytogenetic subtypes quantifying more than 12,000 proteins and 19,000 protein-coding transcripts as well as sensitivity to 528 oncology and investigational drugs. Identify the diacylglycerol-analog bryostatin-1 as a therapeutic candidate in the MEF2D-HNRNPUL1 fusion high-risk subtype ALL

**Table 2:** Proteomic studies on ALL in CSF.

Author/Year/Country	Proteomic Approach	Main Results
Priola GM et al. [111] 2015 USA	LC-MS/MS	Protein C Inhibitor (SERPINA5) and heparin cofactor II (SERPIND1) changed over the course of therapy. Antithrombin III (ATIII) and plasminogen (PLMN) levels decreased expression over time in one child with CNS thrombosis.
Guo L et al. [112] 2019 China	LC-MS/MS	51 proteins identified, 32 up-regulated and 19 down-regulated including TIMP1, LGALS3BP, A2M, AHSG, FN1, HRG,ITIH4, CF I, C2, C4A.
Mo F et al. [113] 2019 China	LC-MS/MS	428 unique proteins identified, 10 altered proteins during treatment
Yu Q et al. [71] 2020 USA	4-plex N,N dimethyl leucine (DiLeu) isobaric labeling strategy and MS	63 proteins were significantly altered

## Data Availability

Data sharing is not applicable to this article.
